# Crystal Structure of Flagellar Export Chaperone FliS in Complex With Flagellin and HP1076 of *Helicobacter pylori*

**DOI:** 10.3389/fmicb.2020.00787

**Published:** 2020-05-19

**Authors:** Wendy Wai-Ling Lam, Kailei Sun, Huawei Zhang, Shannon Wing-Ngor Au

**Affiliations:** Center for Protein Science and Crystallography, School of Life Sciences, Faculty of Science, Chinese University of Hong Kong, Shatin, Hong Kong

**Keywords:** bacterial motility, flagellar filament, export chaperone, *Helicobacter pylori*, secretion system

## Abstract

Functional flagella formation is a widespread virulence factor that plays a critical role in survival and host colonization. Flagellar synthesis is a complex and highly coordinated process. The assembly of the axial structure beyond the cell membrane is mediated by export chaperone proteins that transport their cognate substrates to the export gate complex. The export chaperone FliS interacts with flagellin, the basic component used to construct the filament. Unlike enterobacteria, the gastric pathogen *Helicobacter pylori* produces two different flagellins, FlaA and FlaB, which exhibit distinct spatial localization patterns in the filament. Previously, we demonstrated a molecular interaction between FliS and an uncharacterized protein, HP1076, in *H. pylori*. Here, we present the crystal structure of FliS in complex with both the C-terminal D0 domain of FlaB and HP1076. Although this ternary complex reveals that FliS interacts with flagellin using a conserved binding mode demonstrated previously in *Aquifex aeolicus*, *Bacillus subtilis*, and *Salmonella enterica* serovar Typhimurium, the helical conformation of FlaB in this complex was different. Moreover, HP1076 and the D1 domain of flagellin share structural similarity and interact with the same binding interface on FliS. This observation was further validated through competitive pull-down assays and kinetic binding analyses. Interestingly, we did not observe any detrimental flagellation or motility phenotypes in an *hp1076*-null strain. Our localization studies suggest that HP1076 is a membrane-associated protein with a cellular localization independent of FliS. As HP1076 is uniquely expressed in *H. pylori* and related species, we propose that this protein may contribute to the divergence of the flagellar system, although its relationship with FliS remains incompletely elucidated.

## Introduction

The bacterial flagellum is a supra-molecular complex resulting from the self-assembly of more than 20 different proteins. The overall complex comprises three sub-structures: the basal body, the hook and the filament (Blair, 2003; Macnab, 2004; Berg, 2008). The flagellar assembly process requires hierarchical flagellar gene transcription and checkpoint mechanisms to ensure that these three sub-structures are constructed with precise stoichiometry and in a coordinated order (Chevance and Hughes, 2008). The transport of flagellar proteins across the inner membrane via the flagellar export apparatus is necessary for the construction of the axial structure comprising the hook and the filament (Minamino, 2014, 2018). In *Escherichia coli* and *Salmonella* spp., the cytosolic export chaperone proteins FlgN, FliS and FliT bind specifically to hook junction proteins (FlgK and FlgL), flagellin (FliC) and the filament cap protein (FliD), respectively (Auvray et al., 2001; Bennett et al., 2001; Aldridge et al., 2006). Upon binding, these chaperones direct their substrates to the sorting platform of the export apparatus.

The flagellar filament comprises tens of thousands of FliC subunits that assemble into a helical structure with an approximate length of 15 μm. FliC has an approximate length of 500 amino acid residues and contains four domains: D0, D1, D2, and D3 (Samatey et al., 2001; Beatson et al., 2006). X-ray crystallography and cryo-electron microscopy studies of the molecular structure of a flagellar filament from *Salmonella enterica* serovar Typhimurium revealed that these four domains are arranged in a double-tubular structure (Samatey et al., 2001; Yonekura et al., 2003). The conserved D0 and D1 domains form the inner core of the filament, while the variable D2 and D3 domains form the outer core. The filament contains a 2-nm-wide central hydrophilic channel that is composed mainly of D0 domains and is used to transport unfolded FliC molecules to the distal end. In gram-negative β- and γ-proteobacteria, FliC also serves as a pathogen-associated molecular pattern that is recognized by Toll-like receptors (TLRs) on host innate immune cells (Hayashi et al., 2001; Andersen-Nissen et al., 2005; Song et al., 2017). The interaction between the D1 domain of FliC and the ectodomain of TLR5 was determined (Yoon et al., 2012; Song et al., 2017; Ivičak-Kocjan et al., 2018). Recent mutagenesis studies reported that the D0 domain also contributes to TRL5 activation (Forstnerič et al., 2017; Song et al., 2017).

The export chaperone FliS plays a key role in the assembly of the flagellar filament (Auvray et al., 2001; Ozin et al., 2003). FliS deficiency results in a shorter filament with impaired motility (Yokoseki et al., 1995). FliS binds to the C-terminal disordered region of FliC and directs the latter protein to secretory pathways by interacting with the transmembrane export gate protein FlhA (Bange et al., 2010; Xing et al., 2018). FliS also regulates flagellar gene expression by binding to and suppressing the secretion of the anti-sigma factor FlgM (Yokoseki et al., 1996; Xu et al., 2014). Previous studies have revealed the structural basis of the FliS-FliC interaction in *Aquifex aeolicus* and *Bacillus subtilis*, as well as the FlhA-FliS-FliC interactions in *S.* Typhimurium (Evdokimov et al., 2003; Altegoer et al., 2018; Xing et al., 2018). The 40 C-terminal residues of FliC form three helices that wrap around the core of FliS, which has a four-helix bundle structure, in an extended, horseshoe-like conformation. The interaction of FliS and FliC resembles the substrate recognition mechanisms observed in other type III secretion systems. The docking of FliC to the export gate complex is mediated mainly by the interaction between the N-terminal helix of FliS and the hydrophobic cleft of the FlhA cytoplasmic domain, with no direct interaction between FliC and FlhA. A recent genetic study further proposed that FliC must be unfolded prior to export. Moreover, the docking of FliC at FlhA, which is mediated by the FliS-FlhA interaction, facilitates the unfolding of FliC for rapid export.

*Helicobacter pylori* is a stomach-colonizing epsilon-proteobacteria present in approximately half of the global human population (Polk and Peek, 2010). *H. pylori* infection has been recognized as the major cause of gastritis and the primary risk factor for gastric cancer. Genetic and structural studies have increasingly suggested that *H. pylori* has evolved distinct mechanisms of flagellar motility that are essential for gastric colonization (Galkin et al., 2008; Chen et al., 2011; Minamino and Imada, 2015). For example, *H. pylori* expresses multiple chemoreceptors and chemotaxis-coupling proteins CheVs, an additional structural protein FliY in the motor switch complex and non-flagellar proteins associated with the regulation of motor function (Lowenthal et al., 2009; Lertsethtakarn et al., 2011; Zhang et al., 2017; Lam et al., 2018). The flagellar filament of *H. pylori* comprises two types of flagellins, FlaA and FlaB (Suerbaum et al., 1993; Josenhans et al., 1995). FlaA is the major structural component, whereas FlaB is localized proximal to the hook. Both types of flagellin contain approximately 510 amino acid residues and share an overall sequence identity of approximately 60%. The sequences of both the D0 and D1 domains are highly conserved, as observed in other flagellin homologs. Mutant strains defective in FlaA and FlaB expression exhibit irregular flagella formation and reduced colonization abilities. Interestingly, an extensive sequence similarity search did not identify any FlgN and FliT homologs in the *H. pylori* genome, suggesting that FliS may function as a general chaperone with broad substrate specificity. In a previous study, we confirmed an uncharacterized protein, HP1076 as a novel interacting partner of FliS and resolved the molecular structure of the binary complex (Lam et al., 2010). HP1076, which has a helix-rich bundle structure, forms an extensive electrostatic interaction with FliS via helical stacking. HP1076 also possesses *in vitro* co-chaperone activity and enhances FliS folding. Although the biological importance of HP1076 is unclear, its expression has been shown to be RpoN-dependent (Niehus et al., 2004). A previous report of the upregulation of *hp1076*, *flaB*, and *flgE* in a hook-length control protein *fliK* mutant strain (Douillard et al., 2009) suggests that HP1076 may be involved in motility.

Despite our increased knowledge of the structural and functional roles of FliS, the molecular associations within the FliS-FlaA/FlaB interaction in *H. pylori* remain unclear. Here, we report the 2.95 Å crystal structure of the FliS-FlaB-HP1076 ternary complex from *H. pylori*. Notably, the helical conformation of the D0 domain of FlaB differed from that of FliC in *A. aeolicus*, *B. subtilis* and *S.* Typhimurium. Furthermore, HP1076 shares high structural homology with the D1 domain of flagellin and uses the same binding interface on FliS as in *B. subtilis*. In conjunction with biochemical and molecular genetic studies, our results suggest that HP1076 may be a FliS substrate but is not closely and directly associated with the flagellar system.

## Materials and Methods

### Cloning, Expression and Purification

The gene encoding full-length *fliS* (126 amino acids) was amplified from the genomic DNA of *H. pylori* strain 26695 and cloned into the GST expression vector pGEX6p-3. The gene encoding HP1076 (171 amino acids, 19.3 kD), FlaB (514 amino acids, 53.8 kD) and C-terminally truncated FlaB (FlaBc, residues 415–514, 10.8 kD) were cloned into the pAC28m vector, which expresses a 6× His tag at the N-terminal of the recombinant protein. The FlaBc fragment consists of the C-terminal D0 and D1 region based on the multiple sequence alignment with other flagellin homologs. The resulting plasmids pGEX6p-3-*fliS* and pAC28m-*flaBc* were co-transformed into Rosetta 2 cells and grown in LB broth containing selection antibiotics. Protein expression was induced by the addition of 0.2 mM isopropyl β-D-1-thiogalactopyranoside, followed by incubation at 25°C for 18 h. The cells were harvested and lysed by sonication in a lysis buffer containing 10 mM Tris (pH 7.5) and 300 mM NaCl. To purify the FliS-FlaBc complex, the clarified lysate was passed through a glutathione affinity column, after which the GST-tag was removed using Precission protease (GE Healthcare). The eluates containing FliS and His-FlaBc were purified further on a Ni-NTA affinity column and Superdex 75 size-exclusion chromatography column using a buffer containing 10 mM Tris (pH 7.5) and 150 mM NaCl. His-HP1076 was also purified on Ni-NTA affinity and Superdex 75 size-exclusion chromatography columns. To purify the FliS-FlaBc-HP1076 ternary complex, the FliS/FlaBc complex was mixed with His-HP1076 at a molar ratio of 1:1 and incubated on ice for 2 h. The protein mixture was then loaded on a Superdex 75 column. The retrieved fractions containing the FliS-FlaBc-HP1076 complex were concentrated to 6 mg/ml and subjected to crystallization studies.

### *H. pylori* Growth Conditions

The *H. pylori* G27 strain was cultured at 37°C on Columbia blood agar containing 5% defibrinated horse blood and *H. pylori-*selective antibiotics (trimethoprim, amphotericin, vancomycin, cycloheximide, cefsulodin, polymyxin, β-cyclodextrin) under microaerobic conditions (5% CO_2_, 4% O_2_, and 91% N_2_) controlled by AnaeroGen gas packs (Oxoid). *H. pylori* transformant selection cultures also included 5 μg/ml chloramphenicol. Brucella broth containing 10% (v/v) fetal bovine serum (BB10) was used for liquid *H. pylori* culture.

### Development of the *hp1076-*Null and *fliS*-Null Strains

The *H. pylori* G27 deletion strain Δ*hp1076* was created as described in a previous study (Zhang et al., 2017). Briefly, the kanamycin resistance cassette was amplified from a kanamycin-resistant strain of *Campylobacter jejuni* (a gift from KW Ling, CUHK). The PCR product was digested and inserted into a pGEM-*hp1076* plasmid in which *aphA3* was flanked by *hp1076*. The resulting hp1076:*aphA3* fragment was released and cloned into the pBluescript II KS vector, and the insertion was confirmed by sequencing. A similar strategy was used to generate the *fliS*-null strain, except inverse PCR was used to amplify the backbone of the pBluescript vector together with the flanking region of the *fliS* gene. The digested *aphA3* cassette was then inserted into the inverse PCR product pBlueScript-*fliS* to yield the pBS-*fliS*:*aphA3* plasmid in which *aphA3* was flanked by *fliS*. For transformation, the recombinant plasmids on the pBluescript background were methylated using endogenous methylases from a *H*. *pylori* cell lysate according to (Donahue et al., 2000). The methylated plasmids were then introduced into wild-type *H*. *pylori* using natural transformation. Briefly, freshly cultured *H*. *pylori* was mixed with 1 μg of methylated plasmids and cultured in a microaerophilic environment. The cells were then transferred onto blood agar plates supplemented with 10 μg ml^–1^ kanamycin. After 3–4 days, single colonies were picked and sub−cultured on fresh plates. This selection procedure was repeated three times to obtain *hp1076−*null and *fliS*-null strains. Finally, the strains were confirmed by PCR and immunoblotting.

### Immunoblot Detection of HP1076

An anti-HP1076 antibody was produced from the serum of a rabbit that had been immunized with purified recombinant HP1076 protein (Invitrogen antibody production service). The *H. pylori* G27 and Δ*hp1076* strains were grown in Brucella broth for 1 day prior to the detection of HP1076. The cells were pelleted, resuspended in PBS and boiled in the Laemmli sample buffer to yield total cellular proteins. The membranes were incubated with the anti-HP1076 antibody to detect the protein in the lysates.

### Characterization of Flagella Formation by Transmission Electron Microscopy

The *H. pylori* G27, Δ*hp1076* and Δ*fliS* strains were cultured in Brucella broth containing 0.4% cholesterol at 37°C under microaerophilic conditions for 2 days. The cells were then harvested and washed three times with phosphate−buffered saline (PBS). The cells were further resuspended in PBS to an OD600 of 10. Ten-microliter aliquots of the cell suspension were dotted onto a Formvar/Carbon coated grid (TED PELLA) and stained with 0.2% neutral phosphotungstic acid. Excess staining was removed using Whatman paper. Flagellation was examined under a transmission electron microscope (Hitachi H−7650) at 80 kV.

### Characterization of Motility Activity by Soft Agar Assay

The *H. pylori* G27, Δ*hp1076* and Δ*fliS* strains were cultured on Columbia blood agar plate for 3 days. The cells were harvested and resuspended in Brucella broth containing 0.4% cholesterol, and 1-μl aliquots were dotted onto soft agar plates containing Brucella broth, 0.4% cholesterol and 0.35% Bacto agar. The plates were incubated at 37°C for 3 days under microaerobic conditions (5% CO_2_, 4% O_2_, and 91% N_2_) generated by CampyGen sachets (Oxoid) and then the colony diameters were measured and statistically analyzed.

### Cell Fractionation of *H. pylori*

*H. pylori* G27 and *fliS*-null cells were cultured on blood agar plates for 3 days, harvested and washed three times with PBS. The cells were resuspended in the lysis buffer containing 100 mM Tris-HCl (pH 8.0) to an OD600 of 10 and lysed by sonication. The unbroken cells and cell debris were removed by centrifugation at 4°C and 6000 × g for 10 min. The supernatant (i.e., whole cell fraction) was subjected to ultracentrifugation at 4°C and 50,000 × g for 1 hr. The soluble fraction was collected as the cytosolic/periplasmic fraction. The pellet was washed three times, resuspended in the lysis buffer and collected as the total membrane fraction. All fractions were mixed with SDS-PAGE loading buffer and analyzed by SDS-PAGE and immunoblotting.

### Pull-Down Assays and Interaction Studies

*H. pylori G27* cells were lysed by sonication in the buffer containing 20 mM HEPES (pH 7.5), 137 mM NaCl, 27 mM KCl, 5% glycerol, 0.1% Tween-20 and 10 mM DTT. Unbroken cells and cell debris were removed by centrifugation at 4°C and 6,000 × g for 10 min.

The GST-FliS pull down experiment was performed as described previously (Lam et al., 2010). Purified GST and GST-FliS were mixed with HP1076, HP1076ΔN20, or HP1076ΔN20ΔC29 and immobilized to glutathione sepharose. After washing, aliquots of the resin were subjected to SDS-PAGE to confirm the formation of the GST-FliS-HP1076 and GST-FliS-HP1076 ΔN20 complexes. HP1076ΔN20ΔC29 was used as a negative control because this truncated fragment does not interact with FliS (Lam et al., 2010). The resin was incubated with *H. pylori* cell lysate for 3 h and washed three times. The resin beads were suspended in the loading buffer, and protein binding was analyzed by SDS-PAGE.

For the flagellin/HP1076 competitive binding assay, purified GST-FliS was immobilized on glutathione sepharose, washed and aliquoted. After measuring the concentration of protein on the resin, aliquots were mixed with equal amounts of *H. pylori* cell lysate and HP1076 with GST-FliS at different molar ratios (0, 0.5:1, 5:1, and 50:1), followed by a 3-hr incubation. The beads were washed and analyzed by SDS-PAGE. The intensities of the protein bands were quantified using ImageJ software.

### Microscale Thermophoresis

The purified FliS and FliS-FlaBc complex were labeled fluorescently using the NanoTemper Monolith NT Protein Labeling Kit. Next, labeled FliS or FliS-FlaBc (40 nM) was mixed with HP1076 ranging from 6.2 μM to 0.189 nM or 3.83 mM to 117 μM with a volume ratio of 1:1, respectively, in the buffer containing 20 mM Tris (pH 7.5), 150 mM NaCl and 2 mM DTT. The reactions were run at 30% excitation power, 40% MST power and 21.5°C. MO. Affinity Analysis software was used to analyze the data and calculate the dissociation constant (*K*_*d*_) values.

### Crystallization, Data Collection and Structure Determination

Crystals of FliS-FlaBc-HP1076 were obtained using the sitting-drop vapor-diffusion method at 16°C. The buffer contained 3% (v/v) PEG 20000, 0.2 M potassium bromide, 0.2 M potassium thiocyanate and 0.2 M sodium acetate (pH 5.0). The crystals were soaked briefly in the crystallization buffer containing 20% glycerol and were cooled by plunging into liquid nitrogen. The X-ray datasets were collected using beamline 13B1 at the National Synchrotron Radiation Research Center, Taiwan. The datasets were then processed using the iMosflm package (Battye et al., 2011) and were scaled and reduced using SCALA from the Collaborative Computational Project, Number 4 (CCP4) suite (Winn et al., 2011). Crystals of FliS-FlaBc-HP1076 were formed in the P6_1_ space group. The structure was solved by molecular replacement, using FliS and HP1076 from *H. pylori* (PDB ID: 3K1H) as a search model. The molecular replacement program Phaser (McCoy et al., 2007) in the PHENIX suite and data in the resolution range were used, and the refinement and manual rebuilding steps were performed using the programs COOT and Phenix (Adams et al., 2010; Emsley et al., 2010). The statistics of data collection and refinement are summarized in Supplementary Table 1. The coordinates and structure factors of the FliS-FlaBc-HP1076 complex have been deposited in the Protein Data Bank (PDB ID: 6LEA). All figures were prepared using Chimera (Pettersen et al., 2004).

## Results

### Crystal Structure of the FliS-FlaB-HP1076 Ternary Complex From *H. pylori*

The *H. pylori* flagellins FlaA and FlaB each contain four domains, consistent with other flagellin homologs (Beatson et al., 2006). Although the FliS-FliC structures have been resolved in *A. aeolicus*, *B. subtilis* and *S.* Typhimurium (Evdokimov et al., 2003; Altegoer et al., 2018; Xing et al., 2018), recent studies have demonstrated that the C-terminal D0 domain of flagellin is critical to the ability of *H. pylori* to evade TLR-5-mediated host recognition (Forstnerič et al., 2017). Therefore, the atomic details of FlaA/FlaB and the mechanism of binding to FliS are important. We first attempted to express the full-length FlaA and FlaB, as well as a C-terminal fragment encompassing amino acid residues 416–510 of FlaA (FlaAc) and 416–514 of FlaB (FlaBc). Both full-length FlaA and FlaB formed aggregates when expressed and purified individually, which precluded further analysis. We next tested the co-expression of GST-tagged FliS with FlaAc and FlaBc. Notably, the presence of FliS enhanced the solubility of the flagellins, especially FlaBc. The binary complex of FliS-FlaBc was purified using glutathione sepharose and size-exclusion chromatography prior to crystallization studies. However, no protein crystal was obtained. Previously, we reported that HP1076 improved the protein solubility of FliS mutants in which the hydrophobic residues in the interior of the helix bundle had been mutated. We therefore reasoned that HP1076 might also promote the stability of the FliS-FlaBc complex and would enable molecular packing for crystallization. We formed a ternary complex by incubating the FliS-FlaBc complex with HP1076, and purified the new complex using size-exclusion chromatography.

**FIGURE 1:**
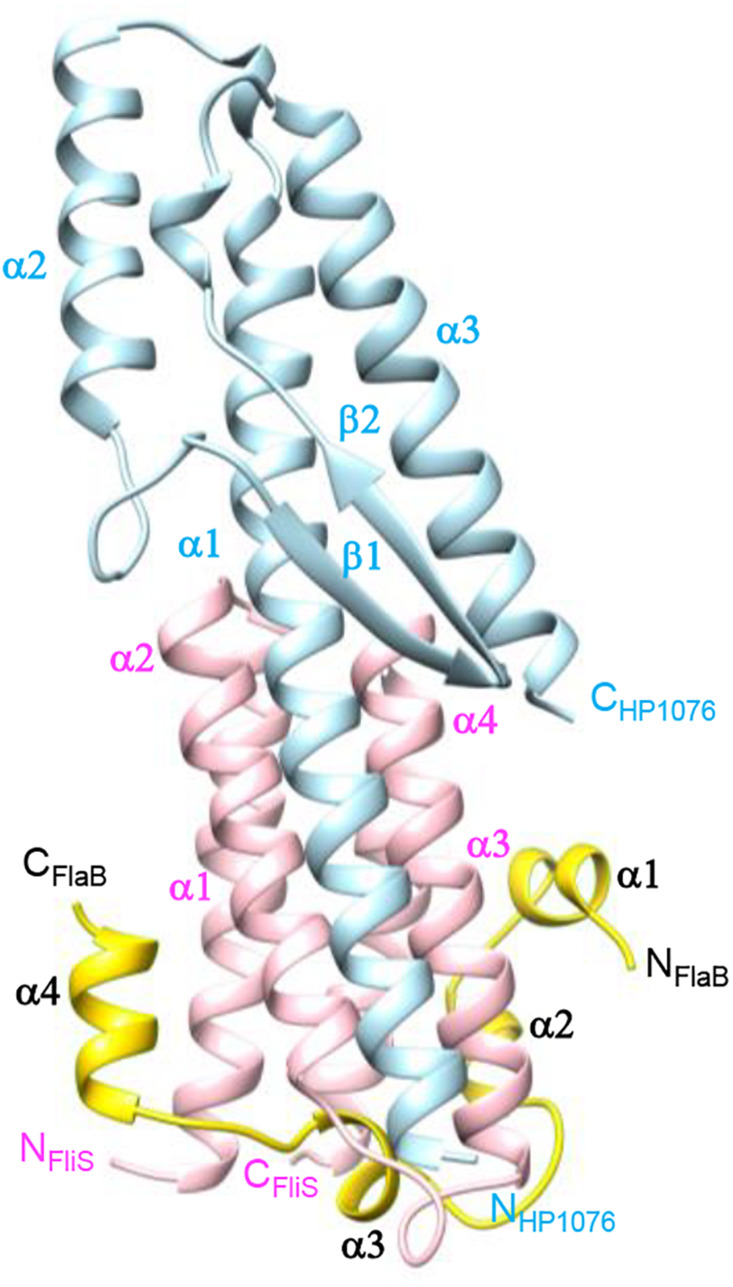
Crystal structure of FliS in complex with FlaB_*C*_ and HP1076. FlaB_*C*_ (yellow) binds exclusively to FliS (pink), while HP1076 (blue) interacts with FliS via the packing of helices α1 of HP1076 and α2–α3 of FliS.

The FliS-FlaBc-HP1076 complex was crystallized in the space group P6_1_, with two ternary complexes per asymmetric unit. The crystal structure was resolved at 2.95 Å with an *R* factor and *R*_*free*_ value of 19.0% and 24.7%, respectively (Figure 1, Supplementary Table 1). The two FliS-FlaBc-HP1076 complexes in the asymmetric unit were virtually identical. The final structure comprised residues 18–122 of FliS, residues 476–514 of FlaB and residues 22–147 of HP1076. The electron density map was clearly defined throughout the structure, except for the regions comprised of residues 1–17 and 123–126 of FliS, residues 416–475 of FlaBc and residues 1–21 and 148–171 of HP1076. FliS formed a four-helix up-and-down bundle structure that resembled its apo-form (PDB ID 3IQC), with a root-mean-square deviation (rmsd) of 0.50 Å. The side chains of residues Leu23, Tyr28, Arg33, Arg56, Glu63, Asn108, and Glu119 adopted a different rotamer conformation to accommodate the binding of FlaBc. Regarding HP1076, superimposition upon our previously solved FliS-HP1076 complex (PDB ID:3K1I) (Lam et al., 2010) yielded an rmsd value of 0.67 Å, with no significant structural difference.

**FIGURE 2:**
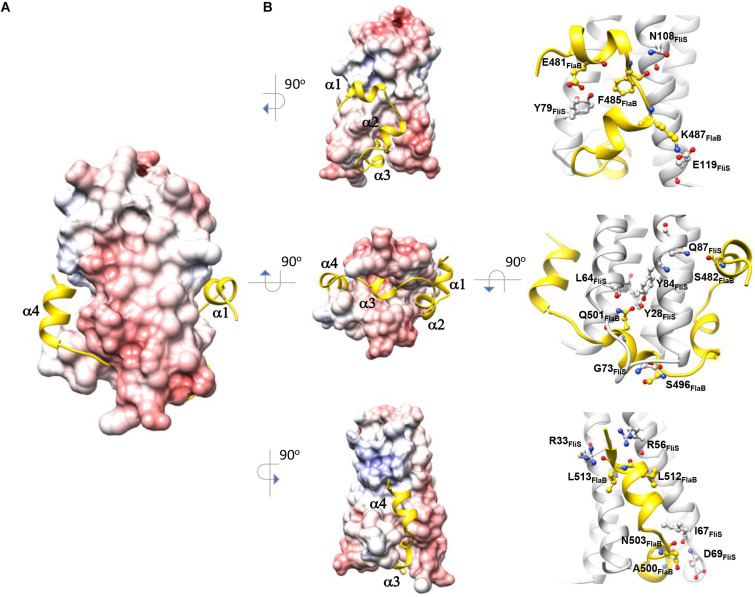
Molecular interaction of FliS and FlaB_*C*_. **(A)** The molecular surface of FliS is colored according to the electrostatic potential, with a contour level of ±10 kT. FliB_*C*_ is represented as a cartoon and colored in yellow. **(B)** The three interfaces of FliS-FliB_*C*_ are drawn. Residues that form interactions via salt bridges and hydrogen bonds are highlighted.

The structure of FlaBc comprised four helices surrounding the core of the FliS helix bundle (Figures 1, 2). FlaBc Helix α1 originated from the last two residues of the spoke region before the D0 domain. Helices α1 and α2 were oriented in a half-opened hairpin structure and interacted with helices α3 and α4 of FliS. Helix 3 was embedded in the flagellar binding pocket, which was consistent with other FliS-FliC structural models (Evdokimov et al., 2003; Altegoer et al., 2018; Xing et al., 2018). Helix 4 was located near the faces of helices α1 and α2 of FliS.

### Molecular Interface of FliS and FlaBc

**FIGURE 3:**
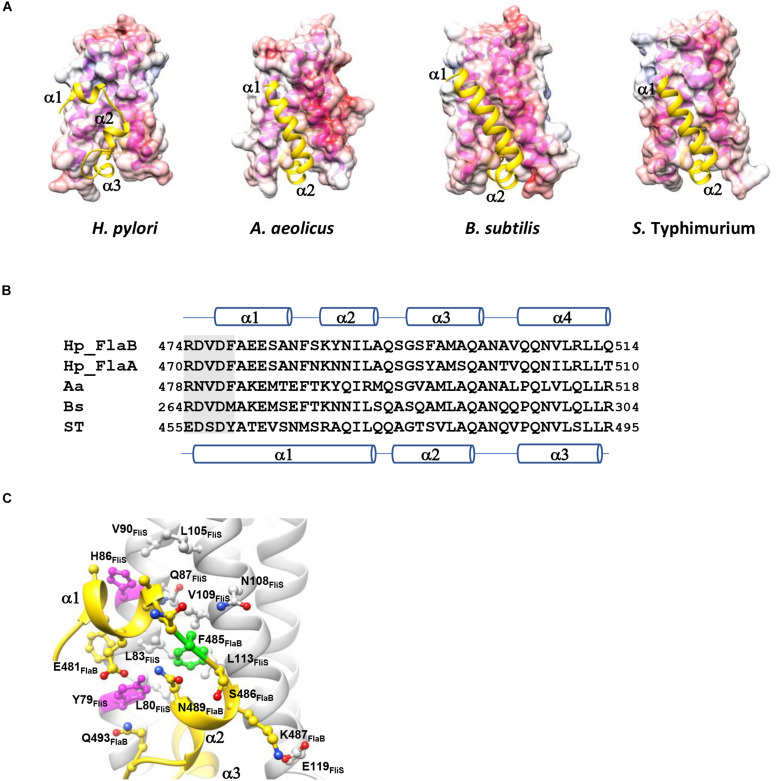
Comparison of the FliS-flagellin binding interfaces in different bacterial species. **(A)** The molecular surface of FliS is colored according to the electrostatic potential, with a contour level of ±10 kT. FlaB_*C*_/FliC are represented by cartoons and colored in yellow. FliS-FliC models of *A. aeolicus*, *S.* Typhimurium and *B. subtilis* were retrieved from PDB ID (1ORY, 6CH3, and 6GOW, respectively). Only the D0 domains of FlaB/FliC are shown. **(B)** Sequence alignment of the spoke region (shaded) and D0 domain in the C-terminal end of flagellin. The secondary structures of FlaB of *H. pylori* and of FliC of *S.* Typhimurium are indicated above and below the sequence, respectively. **(C)** The variable residues Tyr79 and His86 (magenta) shape the FlaB binding groove on FliS. Phe485 of FlaB is buried in the hydrophobic pocket of FliS (highlighted in green).

The binding interface of FliS and FlaBc was analyzed using PDBePISA (Krissinel and Henrick, 2007). This heterodimer contained an extensive buried interfacial area of approximately 1,500 Å^2^ per molecule. The interactions of FliS and FlaBc were stabilized by extensive hydrogen bonding between the side chains and main chains, including Tyr28_*FliS*_: Gln501_*FlaBc*_, Arg33_*FliS*_:Leu513_*FlaBc*_, Arg56_*FliS*_:Leu512_*FlaBc*_, Leu64_*FliS*_:Gln501_*FlaBc*_, Ile67_*FliS*_:Asn503_*FlaBc*_, Asp69_*FliS*_: Ala500_*FlaBc*_, Gly73_*FliS*_:Ser496_*FlaBc*_, Tyr79_*FliS*_:Glu481_*FlaBc*_, Tyr84_*FliS*_:Gln501_*FlaBc*_, Gln87_*FliS*_:Ser482_*FlaBc*_ and Asn108_*FliS*_: Phe485_*FlaBc*_, as well as by the salt bridge Glu119_*FliS*_: K487_*FlaB*_ (Figure 2). The residues on the binding interface were highly conserved, suggesting that the interaction of FlaA with FliS would be similar. Notably, the superimposition of FliS-FlaBc on the FliS-FliC complexes from *A. aeolicus*, *B. subtilis*, and *S.* Typhimurium revealed structural differences. One FliS-FliC binding interface consisted of helical stacking between the α1 of FliC and helices α1 and α2 of FliS. In FliC, however, the extended helix α1 is separated into two distinct helices (α1 and α2), whereas this structure was folded in a hairpin in FlaBc (Figures 3A,B). We also examined whether crystal packing could induce the structural variation observed in FlaBc. Although we observed a symmetrical equivalent next to helix α2 of FlaBc, molecular packing only involves helix α2, and the solvent environment was sufficiently large to accommodate an extended helical structure (Supplementary Figure 1). Given the relatively high structural and sequence homology of the D0 domain of FliC across other bacterial species, it is interesting that the helical conformation of FlaB changed upon interaction with FliS in *H. pylori*. As the hinge of the hairpin did not contain a glycine or proline residue, we speculated that the hairpin fold was guided by the groove generated by the variable residues Tyr79 and His86 of FliS (Figure 3C). The insertion of Phe485 of FlaB into the hydrophobic pocket of FliS may further promote the binding of the hairpin structure to FliS.

### HP1076 and FliC Share a Common Docking Site on FliS

**FIGURE 4:**
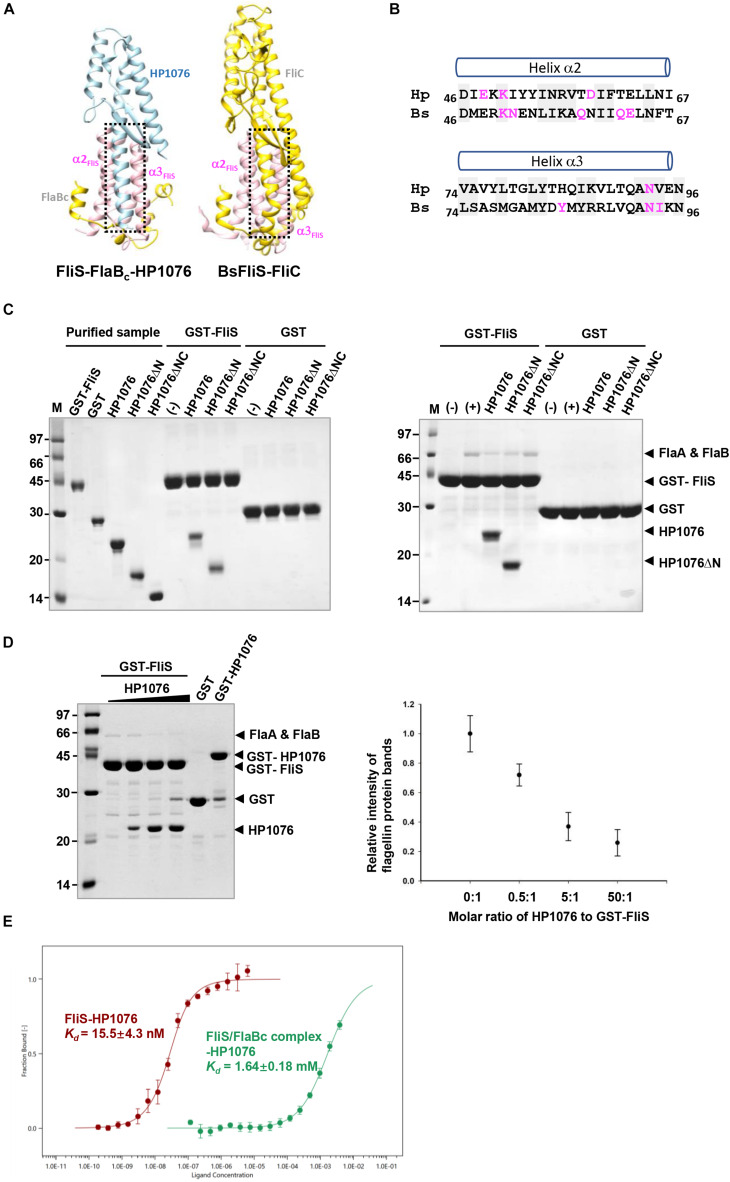
HP1076 competes with FliS to bind flagellin. **(A)** The HP1076-FliS and the FliS-flagellin interaction in *B. subtilis* (PDB ID. 6GOW) use the same FliS binding interface. FliS, flagellin and HP1076 are shown in pink, yellow and blue, respectively. **(B)** The amino acid sequence alignment of helices α2 and α3 of FliS from *H. pylori* (Hp) and *B. subtilis* (Bs). Residues that form hydrogen bonds with HP1076/flagellin are highlighted in magenta. Highly conserved residues are shaded. **(C)** Pull-down assays were performed using purified GST-FliS (with/without HP1076) and an *H. pylori* lysate. (Left) The interactions of FliS with full-length HP1076 and two truncated fragments were examined. HP1076ΔN refers to HP1076ΔN20 and HP1076ΔNC refers to HP1076ΔN20ΔC29. A control experiment lacking HP1076 is marked with (–). Consistent with our previous study (Lam et al., 2010), the deletion of approximately 20 residues from the N-terminal and C-terminal ends of HP1076 abolished the interaction with FliS. (Right) Pull-down assays were performed using GST-FliS and an *H. pylori* lysate. As indicated, full-length HP1076 or a truncated fragment was mixed with beads containing immobilized GST/GST-FliS prior to the addition of lysate. The protein bands indicated by asterisks were excised and identified as flagellin FlaA and FlaB by mass spectrometry. A control experiment lacking the *H. pylori* lysate is marked with (–). Control experiments containing the *H. pylori* lysate but not HP1076 are marked as (+). **(D)** HP1076 and FlaA/FlaB competitively bind FliS. (Left) *H. pylori* lysates were mixed with different amounts of HP1076 and incubated with GST-FliS. Molar ratio of HP1076 to GST-FliS from left to right: 0, 0.5:1, 5:1, and 50:1. GST and GST-HP1076 controls were included. (Right) The relative intensity of flagellin protein bands were quantified and plotted. When GST-FliS was saturated by HP1076, the binding of flagellin was about 1/5 to 1/4 of that when there was no HP1076 binding. **(E)** Microscale thermophoresis assay indicated the affinity between HP1076 and FliS-FlaBc complex was much weaker than that between HP1076 and FliS.

A previous study demonstrated similarities in the fold of HP1076 with those of a flagellin homologue, hook associated protein and FliS (Lam et al., 2010). Given the increasing number of structures deposited into the Protein Data Bank in the last decade, we decided to re-analyze the structural homology of HP1076 using the DALI server (Holm and Rosenström, 2010). The top hits from our homology search included the flagellin from *S.* Typhimurium and *B. subtilis* in complex with TLR-5 PDB ID: 5GY2; Z-score: 11.8; rmsd: 2.4 and PDB ID: 3V47: Z-score 11.8; rmsd 2.2, respectively) and flagellin (PDB ID: 4NX9; Z-score: 11.4; rmsd: 2.4). Superimposition of the structures of HP1076 and FliC revealed that both interacted with helix α2–α3 of FliS (Figures 4A,B). However, these proteins formed hydrogen bonds with FliS at different residues, whereas Lys50 and Asn93 were the only common residues. Given the high structural similarity of HP1076 with the flagellin D1 domain and the FliS binding interface, we hypothesized that HP1076 and FlaA/B may competitively bind to FliS in *H. pylori.* To test this hypothesis, we performed pull-down experiments using GST-FliS and *H. pylori* lysates and included two truncated HP1076 fragments, HP1076ΔN20 (with the deletion of 20 residues from the N-terminus) and HP1076ΔN20ΔC29 (with the deletion of 20 and 29 residues from the N- and C-terminus, respectively), as well as full-length HP1076. Notably, HP1076ΔN20 retains the ability to interact with FliS, whereas HP1076ΔN20ΔC29 exhibited a defective ability to bind to FliS (Lam et al., 2010). We first validated the interaction between FliS and HP1076 before adding the *H. pylori* lysate (Figure 4C). In the absence of HP1076, the GST-FliS pull-down assay yielded a protein band with a molecular weight of approximately 66 kDa. Mass spectrometry was used to determine that this band contained FlaA and FlaB (Supplementary Figure 2). However, the intensity of this band was reduced when the pull-down assays were performed in the presence of HP1076 and HP1076ΔN20. Yet, the addition of HP1076ΔN20ΔC29 to the pull-down assay did not affect the FlaA/FlaB band intensity. We repeated the pull-down assays by titrating different molar ratios of HP1076 to demonstrate further that HP1076 and FlaA/FlaB competitively bind FliS (Figure 4D). The results indicated that the interaction between FliS and FlaA/FlaB was hindered in the presence of HP1076 in a concentration-dependent manner. Furthermore, we performed microscale thermophoresis assays to compare the binding affinities between FliS and HP1076 and between the FliS-FlaBc complex and HP1076 (Figure 4E). The *Kd* of the interaction between FliS and HP1076 was approximately 15 nM. However, the *Kd* of the interaction between FliS-FlaB and HP1076 increased by approximately 10^5^-fold to 1.6 mM. Taken together, these results suggest that HP1076 and FlaA/FlaB share a binding interface on FliS.

### HP1076 Is a Membrane-Associated Protein

**FIGURE 5:**
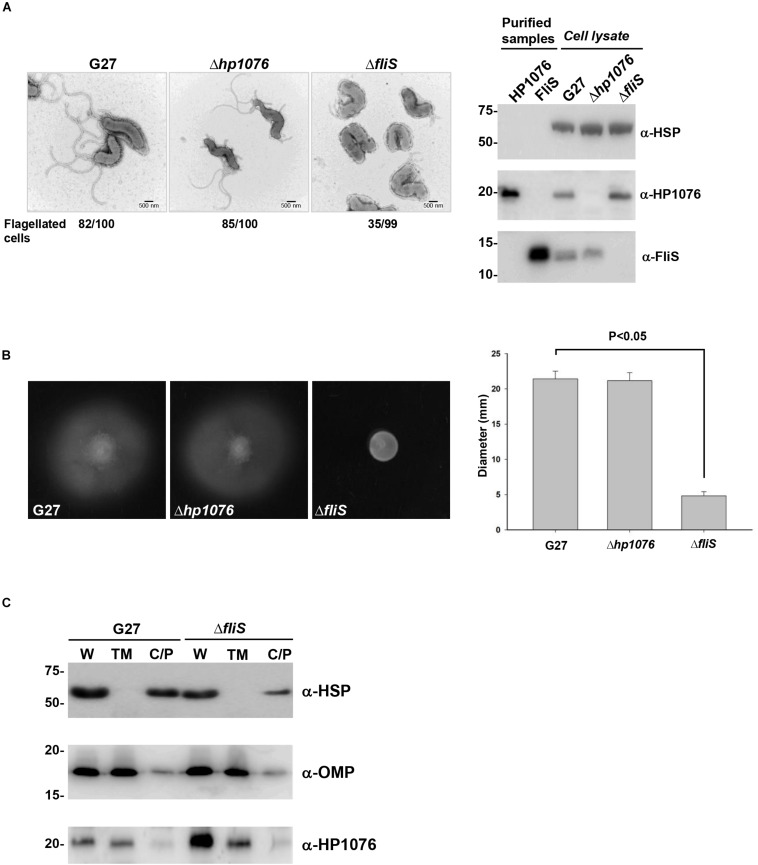
*H. pylori* G27 *hp1076*-null and *fliS*-null strains were constructed and examined. **(A)** (Left) Flagellation in the *H. pylori* G27, *hp1076*-null and *fliS*-null strains was examined by negative staining and transmission electron microscopy. Scale bar = 500 nm. (Right) The G27 *hp1076*-null and *fliS*-null strains were validated by immunoblotting. **(B)** Soft agar motility assay. (Left) Representative images of the diameters of wild-type, *hp1076*-null and *fliS*-null colonies measured after 3–4 days. (Right) The average colony diameter of the *fliS*-null strain was 4.83 ± 0.58 mm (*n* = 12), and was significantly smaller than the diameters of the wild-type (21.42 ± 1.08 mm, *n* = 12) and *hp1076*-null strains (21.17 ± 1.11 mm, *n* = 12). **(C)** Cell fractionation analysis of the *H. pylori* G27 WT and *fliS*-null strains. The whole cell fraction (W), total membrane fraction (TM) and cytosolic/periplasmic fraction (C/P) were blotted using specific antibodies. Heat shock protein (HSP) was used as the marker for the cytosolic/periplasmic fraction, and outer membrane protein (OMP) was used as the marker for the total membrane fraction. HP1076 was mainly detected in the membrane fraction.

To understand the functional importance of HP1076, we performed a molecular genetics study wherein we constructed *hp1076*-null and *fliS*-null mutants of *H. pylori* strain G27. Consistent with the results of previous studies, the deletion of *fliS* led to non-flagellation or shorter flagellar filaments than those in the wild-type strain (Yokoseki et al., 1995). Impaired bacterial motility was also observed in the *fliS*-null strain (Figures 5A,B). However, the disruption of *hp1076* had no effect on flagellation, and the *hp1076*-null and wild-type strains exhibited similar swarming behaviors, suggesting that HP1076 may not be associated directly with flagellar synthesis and export. We further investigated the functional importance of HP1076 in *H. pylori* by examining its cellular localization. We performed an immunoblot analysis of the various cell fractions, using heat shock protein and outer membrane protein as cytosolic and membrane protein markers, respectively. We detected HP1076 predominantly in the membrane fraction (Figure 5C). We also performed a liquid chromatography–mass spectrometry analysis (in duplicate) of the cellular localization of HP1076 to profile the proteins in the membrane and cytosolic/periplasmic fractions extracted from *H. pylori* G27. Notably, HP1076 peptides were identified solely in the membrane fraction (data not shown). Because HP1076 forms a stable complex with the export chaperone FliS, we hypothesized that FliS may be required for the membrane localization of HP0176. We repeated the cell fractionation assays using a *fliS*-null mutant strain (Figure 5C). In the absence of FliS, HP1076 did not accumulate in the cytosol. These findings indicate that the membrane association of HP1076 is independent of FliS.

## Discussion

The bacterial flagellum is a supramolecular complex that contributes to motility. It is also recognized as a virulence factor for colonization and infection in pathogenic bacteria. Although the flagellum is formed via self-assembly, the biosynthesis of the components is regulated by a transcriptional hierarchy that coordinates the expression of flagellar genes with the assembly of the different sub-structures (Chevance and Hughes, 2008; Minamino, 2014, 2018). Flagellar filament is a helical tubular structure that is made up of many thousands of flagellin subunits. The export chaperone FliS is required for the secretion of flagellin monomers through a narrow central channel across the cellular membrane and the subsequent synthesis of the axial structure (Auvray et al., 2001; Ozin et al., 2003). Recently, proposed models of the flagellar system have been based on genetic and structural studies conducted in *E. coli* and *S.* Typhimurium (Minamino, 2014, 2018). The cryo-EM structure of the *S.* Typhimurium flagellar filament reveals the molecular staggering of 11 protofilaments each consisting of four structural domains designated D0-D3 (Yonekura et al., 2003). Helical stacking of the conserved D0 and D1 domains makes up the inner and outer tubes of the filament, respectively. The hypervariable D2 and D3 domains are surface exposed. The N-terminal and C-terminal D0 segments that form coiled-coil interaction are disordered in solution. The chaperone FliS binds to the C-terminal disordered segment of the D0 domain and helps to dock the flagellin to the export gate complex (Bange et al., 2010; Xing et al., 2018). A recent study has proposed that binding of FliS to FlhA also promotes efficient unfolding of flagellin for rapid export (Furukawa et al., 2016).

Although most flagellar proteins in *H. pylori* are homologous to those in other bacterial species, increasing evidence suggests that the flagellar system itself differs from those used in model microorganisms such as *E. coli* and *S.* Typhimurium (Galkin et al., 2008; Chen et al., 2011; Minamino and Imada, 2015). It therefore provides a good model to compare the general principles of flagellar assembly across different bacterial species. Previously, we reported the structure of FliS and its complex with a novel interacting protein HP1076 with no known biological function (Lam et al., 2010). In this study, we determined the molecular structure of FliS in complex with the minor flagellin FlaB and HP1076 in *H. pylori*. Unlike FliC in *B. subtilis* and *S.* Typhimurium, both the major flagellin FlaA and the minor flagellin FlaB are insoluble when overexpressed in *E. coli*. Stable proteins were only obtained when we co-expressed FliS with the FlaA/FlaB containing the C-terminal D0 and D1 segments. Interestingly, crystallization of the complex required the presence of HP1076. The C-terminal D1 segment of FlaB is missing in the ternary complex structure. From the structural model of *S.* Typhimurium flagellin (Samatey et al., 2001; Yonekura et al., 2003), this segment is likely to be disordered in the absence of the N-terminal D1 helix and the hairpin structure. In general, the structure of FliS-FlaBc-HP1076 ternary complex reveals that FliS interacts with FlaB using a conserved binding mode demonstrated previously in *A. aeolicus*, *B. subtilis*, and *S.* Typhimurium (Evdokimov et al., 2003; Altegoer et al., 2018; Xing et al., 2018). However, the helical arrangement of the D0 domain of FlaB in the ternary complex is different. The C-terminal D0 domain of FlaB contains four helices with the first two helices folded in a half-opened hairpin structure and interact with helices α2–α3 of FliS, whereas the last two helices wrap around the opening end and one side of the helix bundle of FliS as in other flagellin homologs. Taken together the structural information from the protofilament and FliS-FliC models (Evdokimov et al., 2003; Altegoer et al., 2018; Xing et al., 2018), it appears that the conformation of the C-terminal D0 segment is dependent on its interacting protein. This segment is in three helices (or four helices in *H. pylori*) when interacting with FliS. However, it exists as one extended helix and stacks with the N-terminal D0 helix in *S*. Typhimurium protofilament. At present, it is unclear whether the conformational differences we observed in *H. pylori* FlaB have any implications in protofilament and/or filament formation. The FliS-FliC model of *B. subtilis* whose flagellin lacks the D2 and D3 domains revealed an additional contact area between the N-terminal D1 domain with FliS (Altegoer et al., 2018). In the FliS-FlaBc-HP1076 model, interaction of HP1076 with FliS mimics the binding of D1 domain with FliS in *B. subtilis*. Although the C-terminal D0 segment of *H. pylori* FlaB shows conformation differences in the complex, we speculate that its D1 domain would display similar interaction with FliS as in the *B. subtilis* model.

Bacterial flagellar filaments also play a critical role in innate immunity in β- and γ-proteobacteria (Hayashi et al., 2001; Andersen-Nissen et al., 2005). In addition to the D1 domain, the D0 domain is required for full TLR5 activation (Yoon et al., 2012; Song et al., 2017; Ivičak-Kocjan et al., 2018). However, β- and ε-proteobacteria are able to evade the TLR5 activation (Andersen-Nissen et al., 2005). In *H. pylori*, both flagellins FlaA and FlaB can evade recognition by TLR5 (Lee et al., 2003). A recent mutagenesis study further proposed that the re-orientation of the D0 domain is required to stabilize the 2:2 interaction between TLR-5 and flagellin but its mechanism is unclear (Forstnerič et al., 2017). Interestingly, the analysis of the crystallographic packing of the FliS-FlaBc-HP1076 complex reveals the inter-molecular stacking of helix α4 of FlaB with its symmetry equivalent (Supplementary Figure 1B). The inter-FlaB interaction involves conserved hydrophobic residues at the distal end of the D0 domain. In *S.* Typhimurium, the corresponding residues were shown to be critical for the TLR-5 activation (Forstnerič et al., 2017). When taken together, the structural model of *H. pylori* FliS-FlaB provides new insights regarding the conformational flexibility of the D0 domain and its ability for this microorganism to evade the TLR-5-mediated host innate immune response.

Our crystal structure FliS-FlaBc-HP1076 complex reveals that HP1076 shares a similar topology with the D1 domain of FliC and a FliS binding interface with FliC. These structural findings are supported by the pull-down results using GST-FliS and *H. pylori* cell lysate that HP1076 hindered the binding of FlaA/FlaB to FliS in a competitive manner. The binding kinetic data demonstrates that the binding affinity of HP1076 to FliS is dramatically reduced when FliS is in complex with FlaBc containing the C-terminal D0 and D1 regions, which further supports that the HP1076 and FlaA/FlaB share the same binding interface on FliS. In addition, we show that HP1076 localizes predominantly at the membrane, but its membrane association is independent of FliS, which indicates that the interaction of FliS and HP1076 has no functional relationship as a chaperone and a substrate. Although the *hp1076*-null mutant shows a flagellation and motile phenotype resembled to those of the wild type, we do not exclude the possibility that HP1076 is involved in the flagellar system. Purified HP1076 was highly soluble and did not exhibit polymerization or aggregation, it is unlikely that itself forms a high-order polymer. The regulation of flagellar gene transcription in *H. pylori* is unique compared with the model microorganisms (Niehus et al., 2004). Three RNA polymerase sigma factors σ^80^, σ^54^ (also called RpoN) and σ^28^ control the transcription of early flagellar genes, middle flagellar structural genes and late flagellar genes, respectively. The RpoN regulon includes flagellar hook subunit *flgE*, hook-filament adaptor proteins *flgK* and *flgL*, and the minor flagellin *flaB*. Interestingly, the expression of *hp1076* is RpoN-dependent and its transcription level is found to be up-regulated together with *flaB* and *flgE* in a *fliK* mutant which displayed impaired motility and polyhook structures (Douillard et al., 2009). Recent interactome studies of *H. pylori* have further identified the binary complex of FliS-HP1076 and thus highlighting the potential functional significance of this heterodimer (Wuchty et al., 2018). Given that HP1076 is uniquely found in epsilon-proteobacteria and adopts a flagellin-like structure, it may play a specific role for the motility adaptation of this microbial species. We speculate that HP1076 may function as a minor regulatory or structural protein of the flagella, in particular for the transcription of hook-associated genes or for the assembly of the hook, respectively. Notably, the crystal structure of HP1076 alone [PDB ID 3K1H] revealed the rearrangement of the N-terminal region from a β-strand to an α-helix conformation upon interacting with FliS (Lam et al., 2010). This strand-to-helix transformation of HP1076 gives a better structural alignment with the D1 domain of flagellin. It is unclear which conformation is adopted by membrane-associated HP1076. In the future, the identification of additional interacting partners of HP1076 would provide further insights regarding the functional importance of this hypothetical protein.

In summary, our data contribute to our understanding of the mode of binding between export chaperone proteins and flagellin, as well as the largely unexplored protein HP1076, in *H. pylori*. Combined with the structural information about FlaB, our findings open a new direction for the studies of the evasion of TLR-5 recognition by *H. pylori* and the broad spectrum of FliS substrates.

## Data Availability Statement

The datasets generated for this study can be found in the Protein Data Bank: accession number 6LEA.

## Author Contributions

WL, KS, HZ, and SA designed and carried out the research. KS and SA analyzed the data. WL, KS, HZ, and SA wrote the manuscript.

## Conflict of Interest

The authors declare that the research was conducted in the absence of any commercial or financial relationships that could be construed as a potential conflict of interest.
