# Supplementary material for: Crystal Structure of Flagellar Export Chaperone FliS in Complex With Flagellin and HP1076 of Helicobacter pylori
Source: Front Microbiol. 2020 May 19;11:787. doi: 10.3389/fmicb.2020.00787 (PMC7248283; doi:10.3389/fmicb.2020.00787)
Supplement: Supplementary file 1 [file Data_Sheet_1.PDF]

Supplemental Table 1. Data collection and refinement statistics of FliS-FlaBc-HP1076 crystal

**Data collection**	
Space group	P 6_1
Cell dimensions	
a, *b*, *c* (Å)	103.33, 103.33, 144.21
α, *β*, *γ* (°)	90.00, 90.00, 120.00
Resolution (Å)	30.62 - 2.95 (3.11- 2.95)
R-meas	0.091 (0.452)
I/σ(I)	16.6 (4.6)
Completeness (%)	90.9 (98.4)
Redundancy	9.7 (9.1)
**Refinement**	
Resolution (Å)	30.62 – 2.95
No. reflections	16113
R_{work}/R_{free}	0.190 / 0.247
No. atoms	4283
Protein	4272
Ligand/ion	-
Water	11
B-factors	
Protein	61.75
Ligand/ion	-
Water	38.39
R.m.s. deviations	
Bond lengths (Å)	0.007
Bond angles (°)	0.986
Ramachandran	96.95/3.05/0

N.B. Statistics for the highest-resolution shell are shown in parentheses.
