# Supplementary figures and images for: Crystal Structure of Flagellar Export Chaperone FliS in Complex With Flagellin and HP1076 of Helicobacter pylori
Source: Front Microbiol. 2020 May 19;11:787. doi: 10.3389/fmicb.2020.00787 (PMC7248283; doi:10.3389/fmicb.2020.00787)

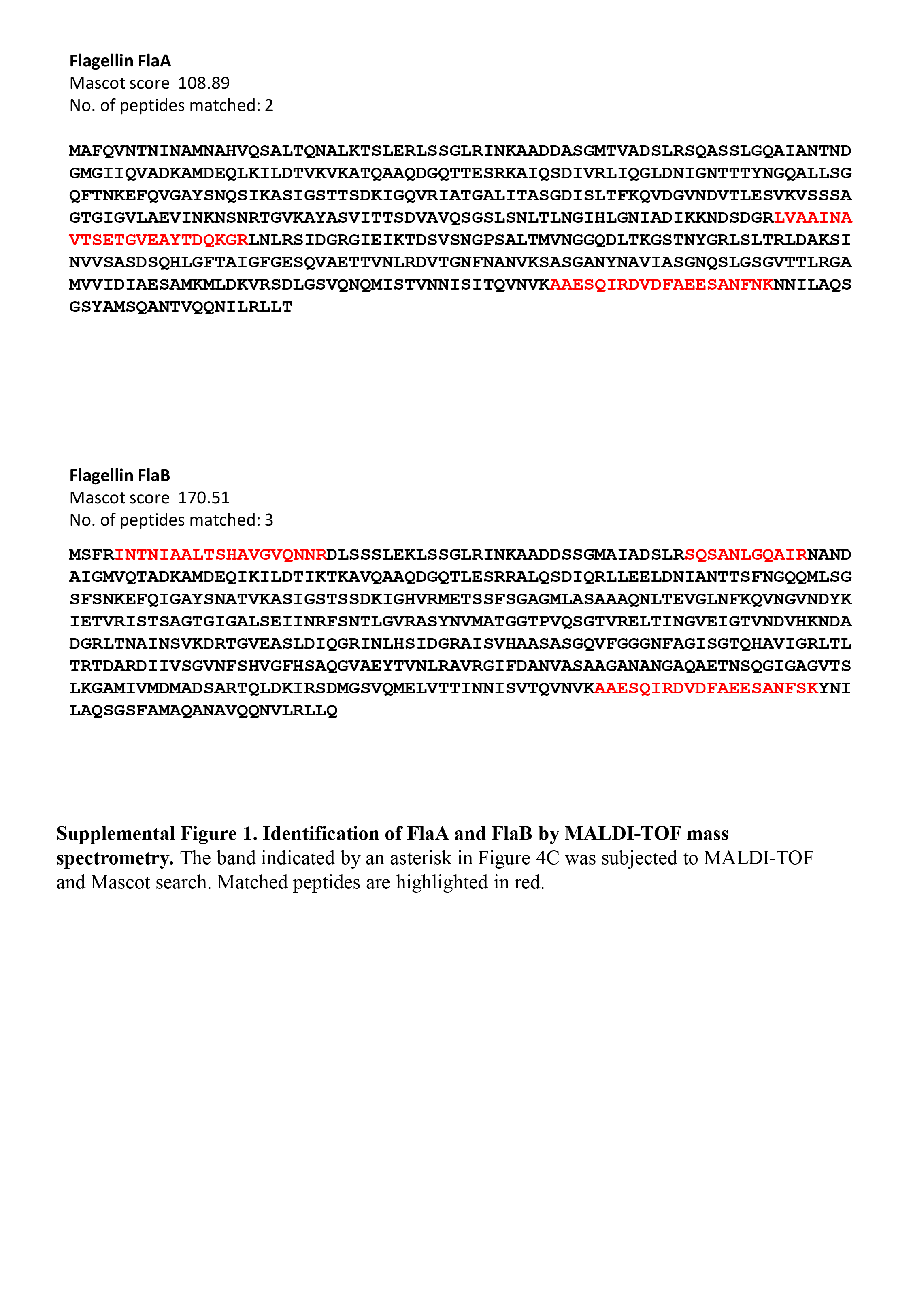

Supplement: Supplementary file 2 [file Image_1.TIF]

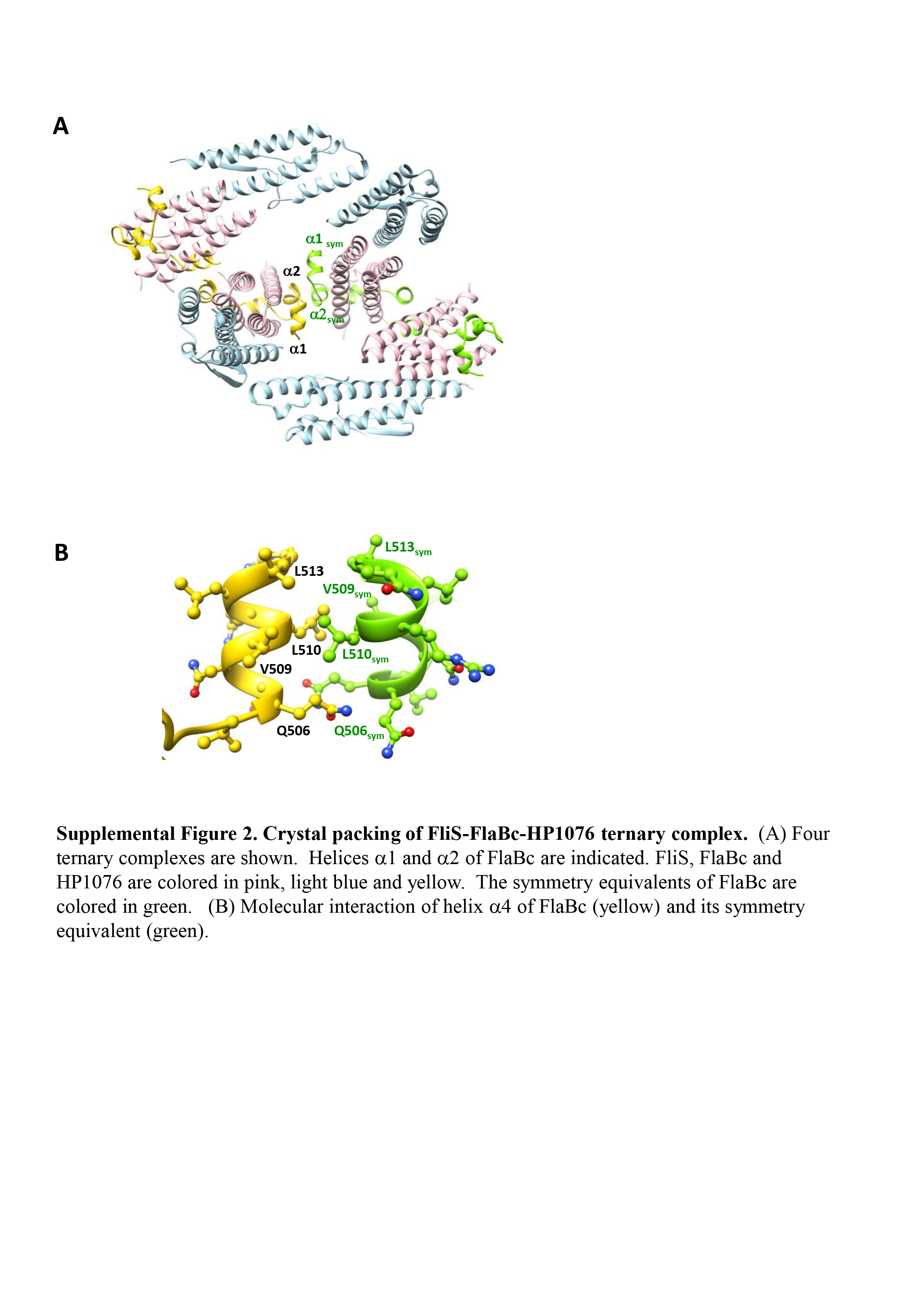

Supplement: Supplementary file 3 [file Image_2.TIF]
